# Positive Edge Effects on Forest-Interior Cryptogams in Clear-Cuts

**DOI:** 10.1371/journal.pone.0027936

**Published:** 2011-11-17

**Authors:** Alexandro Caruso, Jörgen Rudolphi, Håkan Rydin

**Affiliations:** 1 Department of Ecology, Swedish University of Agricultural Sciences, Uppsala, Sweden; 2 Department of Plant Ecology, Uppsala University, Uppsala, Sweden; Umea University, Sweden

## Abstract

Biological edge effects are often assessed in high quality focal habitats that are negatively influenced by human-modified low quality matrix habitats. A deeper understanding of the possibilities for positive edge effects in matrix habitats bordering focal habitats (e.g. spillover effects) is, however, essential for enhancing landscape-level resilience to human alterations. We surveyed epixylic (dead wood inhabiting) forest-interior cryptogams (lichens, bryophytes, and fungi) associated with mature old-growth forests in 30 young managed Swedish boreal forest stands bordering a mature forest of high conservation value. In each young stand we registered species occurrences on coarse dead wood in transects 0–50 m from the border between stand types. We quantified the effect of distance from the mature forest on the occurrence of forest-interior species in the young stands, while accounting for local environment and propagule sources. For comparison we also surveyed epixylic open-habitat (associated with open forests) and generalist cryptogams. Species composition of epixylic cryptogams in young stands differed with distance from the mature forest: the frequency of occurrence of forest-interior species decreased with increasing distance whereas it increased for open-habitat species. Generalists were unaffected by distance. Epixylic, boreal forest-interior cryptogams do occur in matrix habitats such as clear-cuts. In addition, they are associated with the matrix edge because of a favourable microclimate closer to the mature forest on southern matrix edges. Retention and creation of dead wood in clear-cuts along the edges to focal habitats is a feasible way to enhance the long-term persistence of epixylic habitat specialists in fragmented landscapes. The proposed management measures should be performed in the whole stand as it matures, since microclimatic edge effects diminish as the matrix habitat matures. We argue that management that aims to increase habitat quality in matrix habitats bordering focal habitats should increase the probability of long-term persistence of habitat specialists.

## Introduction

Habitat destruction and fragmentation are considered major threats to biodiversity [1]. Land conversion leads to landscapes composed of habitats of variable quality for the organisms, and the creation of boundaries (edges) between habitat types. Edges between ‘focal’ (high quality) and ‘matrix’ (low quality, often human-modified) habitats dominate many landscapes shaped by human land use, and abiotic and biological changes in and near edges (edge effects) are major causes for the impact of fragmentation on the distribution of species [2]. The strength of biological edge effects, and the distance at which they occur are influenced by microclimatic gradients, propagule flow, species interactions, and resource quality and availability across edges [2]–[5]. Since the 1990s, the majority of studies investigating edge effects have concentrated on focal habitats that are negatively influenced by bordering matrix habitats [6]. The possibilities for positive edge effects in matrix habitats bordering focal habitats (e.g. spillover effects) have, however, received little attention.

The consideration of the matrix as uniform nonhabitat is an oversimplification for many species [7]–[8]. In fact, bird, insect, and cryptogam species described as focal habitat specialists have been found to inhabit matrix habitats [9]–[12]. In addition, some bird [13], insect [14] and vascular plant species [15] that occur in matrix habitats are more abundant nearer to focal habitat edges (i.e. positive edge effect in the matrix habitat). Since the matrix may develop into focal habitats through succession, matrix management may be a more efficient conservation strategy than experimenting with size and spatial configuration of remnant focal habitats in fragmented landscapes [7]–[8], [16]. Moreover, the latter is often not a possible strategy. A deeper understanding of ecological patterns and processes in matrix habitats is essential for enhancing landscape-level resilience to human alterations.

Commercial logging is responsible for the fragmentation of European and North American boreal forests [17]–[18]. This transformation of natural forests leads to a landscape dominated by a matrix of young managed stands, and where the focal, late-successional (old-growth) stands are few and isolated. The mature old-growth forest fragments play an important role in hosting species that are dependent on shady and moist forest-interior conditions [19]. Edge habitats between mature and young forests are, however, intermediate to forest-interior and open habitats in terms of e.g. exposure to sun and wind [20]. These microclimatic edge effects further reduce the total area with intact habitat-interior conditions in the fragmented landscape, and directly affect species in both the mature and young stand [2].

Cryptogams (lichens, fungi, and bryophytes) are key components for the maintenance of biodiversity in forest ecosystems. They are important for e.g. nutrient cycling, nitrogen budgets, and as habitats and food for numerous organisms [21]–[23], and many are widely used as indicators of old-growth boreal forests [24]. The occurrence, abundance, diversity, and growth of forest-interior cryptogams are negatively affected by the microclimatic edge effects towards clear-cuts [25]–[28]. In boreal forests, these biological edge effects are, however, weakest in mature forests located to the south of matrix habitats [26]–[27] where the mature forest edge is less exposed to sun and wind [29]–[30]. This suggests that it might be possible for forest-interior species to survive in, or re-colonize, the southern edge of clear-cut habitats bordering mature forests. The re-colonization rate of forest-interior species in clear-cuts could, in turn, be influenced by the local propagule source, since restricted dispersal has been shown to affect cryptogam species distributions [31]–[33].

Clear-cuts hold great amounts of coarse dead wood (e.g. stumps and logs), a substrate that is rare in managed forests [34]. The shortage of dead wood in managed boreal forests is, in fact, a severe threat to the diversity of forest species [19], of which epixylic forest-interior cryptogams are among the most threatened organisms [35]. Thus, the incentive for our study is the notion that matrix management designed to retain and provide suitable substrates for forest-interior cryptogams may have a potential to enhance re-establishment of these species in matrix habitats. From a management point of view, such efforts could be feasible.

We explored the potential of epixylic forest-interior cryptogams (associated with mature old-growth forests) to inhabit young clear-cut forests (“matrix habitats”). Our main aim was to test for edge effects in the young stands, since we envision that sections bordering a mature forest stand (“focal habitat”) should be affected in microclimate and propagule availability. We focused on edges located north of mature forests (i.e. south-oriented edges), and tested for effects of distance from the mature forest on the composition and frequency of forest-interior specialists in the young stand, while accounting for local environment and propagule sources. For comparison, we also surveyed epixylic cryptogams associated with open habitats, as well as generalist cryptogams (with no obvious association with either forest-interior or open habitats).

We hypothesized that if forest-interior species are able to grow in young forests, their frequency of occurrence should increase with decreasing distance to the mature forest (positive edge effect in the matrix). For open-habitat species we instead expected lower frequency of occurrence near the mature forest (negative edge effect). Finally, we hypothesized no effect of distance on the frequency of generalist species.

## Methods

No specific permits were required for the described field study.

### Study sites and species

The study was conducted in the Swedish provinces of Uppland (59°43′N, 17°30′E) and Hälsingland (61°52′N, 16°33′E), situated within the boreonemoral and southern boreal vegetation zone [36], respectively. In each of the two provinces we selected one study region (2 700 and 2 300 km^2^ in Uppland and Hälsingland, respectively). Each region was composed of managed mixed coniferous forests of different successional stages, covering the entire rotation period of about 80–100 years, and with scattered occurrence of mature old-growth forest stands with high conservation values, e.g. woodland key habitats or reserves. Woodland key habitats are small habitat patches that are located in managed forest landscapes, and they are supposed to be of key importance for maintaining biodiversity at the landscape-level [37]. Norway spruce (*Picea abies* (L.) Karsten) and Scots pine (*Pinus sylvestris* L.) dominate the landscape, with scattered occurrence of birch (*Betula pendula* Roth and *B*. *pubescens* Ehrh.) and aspen (*Populus tremula* L.).

We used national databases and expert evaluations to establish a list of epixylic cryptogam species comprising 20 lichen, 14 bryophyte and 15 fungal species (species and sources are listed in Table S1). Fungal species treated in this study are polypores or aphyllophoroid fungi. All selected species have been recorded in the study regions and predominantly occur on coniferous dead wood. We classified the species as either “forest-interior” (32) or “open-habitat” (9) based on their associations with mature canopy-closed or young open forests, respectively. Species with no obvious association with either closed or open habitats were classified as “generalists” (8). Open-habitat and generalist species included only lichens.

We used databases from the Swedish Forest Agency and forest owners to identify dead wood rich mature old-growth stands (focal habitats), and young clear-cut stands (matrix habitats) . The mature stands were woodland key habitats or reserves that had been classified by the Swedish Forest Agency or forest owners as valuable for biological conservation due to their high amounts of dead wood. All mature stands were characterized by a multilayered canopy with a mixture of predominantly Norway spruce and Scots pine, but with higher abundances of birch, aspen, and rowan (*Sorbus aucuparia* L.) than in the young stands. The young stands were even-aged monocultures between 6 and 21 years old, located on flat ground and were representative of the study region in terms of timber productivity and tree species composition (70 – 100% of basal area of Norway spruce).

From a larger pre-selection of sites that fulfilled all criteria mentioned above, we then randomly selected 15 study sites in each of the two study regions. Each site consisted of one young and one mature stand sharing a straight border of at least 200 m. We minimized the effect of a sharp contrast between the stand types by only selecting among study sites where the mature stand was located to the south of the young one. Average stand sizes (sd) were 15.4 (11.6), and 8.4 (7.5) ha for young and mature stands, respectively.

### Sampling and species registration

**Figure 1:**
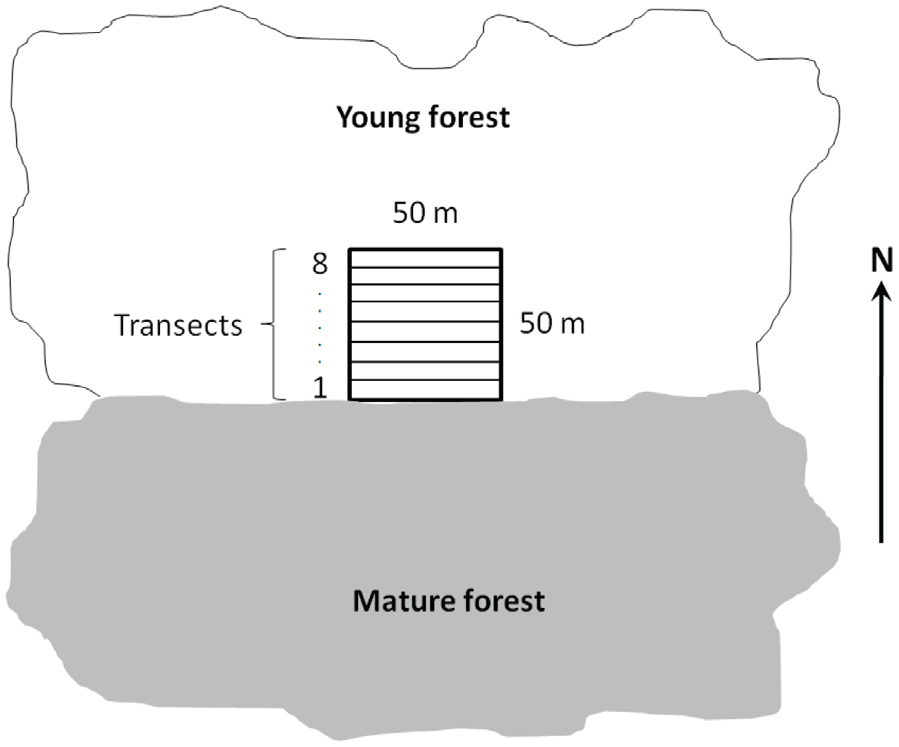
Sampling design. Placement of the 50×50 m plot within the young forest stand at each study site.

The field work was performed during autumn. In each young stand we established a 50×50 m plot (i.e. reaching about two tree heights into the stand) with one side on the border to the mature stand. The plot was centred along the borderline, and divided in eight 6.25 m wide and 50 m long transects aligned parallel to the border (Fig. 1). Within the transects we recorded presence of the cryptogam species on all dead wood objects with a diameter >10 cm (“substrates” hereafter). All species identifications were done in the field. The substrates were classified as either logs or stumps (man-made); no snags (created by natural causes) were found. In total we found 601 logs and 2 297 stumps. We excluded 38 stumps since they were higher than 150 cm and their cut surface area could not be easily surveyed. We also excluded 17 logs since they were present in more than one transect.

### Explanatory variables

For each substrate we recorded three local explanatory variables assumed to affect the occurrence of epixylic cryptogams: area, decay and shade. We measured the average diameter and length or height of logs and stumps, and calculated the surface area using the formulas for a cylinder. For logs we only included 2/3 of the lateral surface area in the calculations to account for the proportion pressed to the ground. We estimated degree of decay by measuring the penetration depth of a sharp pocket knife in different parts of the substrate. The dominating depth was then used to assign the substrate to one of five decay classes: 1) hard, knife penetrating only a few mm, 2) knife penetrating 1–2 cm, 3) knife penetrating 2–5 cm, 4) knife penetrating >5 cm, 5) very soft wood, substrate disintegrates when lifted. Shade was estimated according to 1) exposed to direct sunlight during ≥ 50% of the day, 2) exposed to direct sunlight during<50% of the day, and 3) never exposed to direct sunlight. For the estimation of degree of shade we took into account the number, height, and cardinal point (relative to the stump) of all bushes and trees surrounding the stumps.

To test for influence of local propagule sources for forest-interior cryptogams we spent one hour searching for the species in the adjoining mature stand. We specifically searched substrates with the highest probabilities of species occurrence based on knowledge of the specieś habitat requirements. In the statistical tests we then included as predictors 1) the number of forest-interior cryptogams, and 2) presence/absence of individual forest-interior species in the mature stand for the analyses of forest-interior species as a group and individual forest-interior species, respectively.

To estimate the amount of propagule sources for open-habitat cryptogams we used databases from the Swedish Forest Agency and forest owners, and ArcGis 9.3 to calculate the total area of young stands within a circular buffer zone around each plot. In order to avoid overlapping buffers we set the radius to 500 m. We included stands with a basal area consisting of at least 50% conifers. A pilot study had established that in stands younger than six years, no visible colonizations of epixylic cryptogams could be detected on dead wood created at final felling. In addition, in stands older than 22 years in Uppland and 27 years in Hälsingland (because of the somewhat slower decay process in Hälsingland), stumps and logs were highly decayed and often completely covered by non-epixylic bryophytes. Based on these observations, we defined an age span for stands as potential propagule sources for open-habitat species: the minimum age was set to six years and the maximum to 16 years older than the focal clear-cut in Uppland, and 21 years older in Hälsingland.

### Statistical analysis

The main analyses were done at the transect level (i.e. each transect was used as an observation). We calculated the proportion of substrates in one transect occupied by a given species and used that value, called “occupancy”, to represent the frequency of occurrence. For the three species groups (forest-interior, open-habitat, and generalist species) we defined occupancy as the proportion of substrates that held at least one species from the group.We used occupancy, rather than absolute number of species records in one transect, since 1) the number of substrates was correlated with distance from the mature forest (Pearson r = −0.24, *p* <0.001), and 2) only 3 (0.1%), 192 (6.7%), and 49 (1.7%) substrates were inhabited by more than one forest-interior, open-habitat, and generalist species, respectively. All explanatory variables on the local substrate level (i.e. area, decay, and shade) were averaged for each transect.

For the analysis of species composition along the distance from the mature forest, we used individual species occupancies and performed a randomized complete blocks PERMANOVA (permutational multivariate analysis of variance; [38]), with transect number as the fixed factor and stand identity as the blocking factor. We arcsine squareroot transformed species occupancies, as recommended for proportion data [39]. We used Sørensen distance measure, and performed 4 999 permutations. For the graphical illustration of differences in species composition among transects we used non-metric multidimensional scaling (NMS). The calculations of ordination scores were based on average occupancy values (arcsine squareroot transformed) of individual species within each region. Species that occurred in only one transect were excluded. The final, two-dimensional NMS-solution was obtained with 112 iterations using the “slow and thorough’’ autopilot mode (with the Sørensen distance measure), and the final stress ( =  9.5) was significantly lower than 250 randomized runs (*p*<0.05 for the Monte Carlo test). Stress is a measure (on a scale of 0–100) of the difference between the rank order of distances in the data matrix and the rank order of distances in the reduced-dimensional space of the ordination matrix [40]. For both the PERMANOVA and NMS, we used PC-ORD, version 5.31 [39].

We used generalized linear mixed models, with Proc Glimmix in SAS 9.2 to test for effects of distance from the mature stand and explanatory variables on occupancy of 1) species groups, and 2) individual cryptogam species. The following ten species had sufficient number of occurrences to allow us to test for effects of distance on their individual occupancies (i.e. the models converged): Forest-interior – *Absconditella lignicola* Vězda & Pisút, *Anastrophyllum hellerianum* (Nees ex Lindenb.) R.M. Schust, *Antrodia serialis* (Fr.) Donk, and *Trichaptum abietinum* (Dickson: Fr.) Ryvarden; Open-habitat – *Cladonia botrytes* (K.G. Hagen) Willd., and *Mycocalicium subtile* (Pers.) Szatala; Generalists – *Calicium glaucellum* Ach., *C*. *trabinellum* (Ach.) Ach., *Xylographa parallela* (Ach.:Fr.) Fr., and *X. vitiligo* (Ach.) J.R. Laundon. In addition to the transect-level tests, we also tested occurrence probability (presence or absence) of these individual species on individual substrates as the response variable in the generalized linear mixed models.

We assumed binomial distributions, and used a logit link function (logistic regression) for all univariate tests. To account for the hierarchy in the sampling structure we nested stand identity within region and treated it as a random effect since observations (transects or individual substrates) from one stand are not independent from each other.

The list of fixed effects tested included distance from the edge (transect number), region, stand age, proportion of stumps within transects, substrate area, decay, shade and the variables for propagule sources (see *Explanatory variables* above, and Table S2 for mean and range of variables). All fixed variables were checked for cross-correlations prior to the analyses. The highest Pearson correlation coefficients were found between proportion of stumps within transects and substrate area (r = −0.55, *p*<0.001), decay and shade (r = 0.46, *p*<0.001), and decay and stand age (r = 0.40, *p*<0.001). All other Pearson correlation coefficients were <0.35. Substrates in Uppland were more decayed than those in Hälsingland (t = −8.36, *p*<0.001). We log-transformed substrate area since this gave a better model fit as judged by lower AIC-values (Akaike's Information Criterion) in the model building process (see below). Since some species or species groups may respond non-linearly to decay, we also included squared decay.

In the model building process we first assessed the importance of each of the fixed variables by investigating them one by one. We also tested for effects of biologically reasonable two-way interactions. The random effect (stand identity within region) was always included. A multiple starting model was then constructed. It included all variables with the *p*-value of the associated slope parameter<0.2. The starting model was simplified following a stepwise procedure based on ecological knowledge of the system studied [41] and AIC-values for model selection.

## Results

In total we found 21 forest-interior, 6 open-habitat, and 8 generalist cryptogam species (Table S1). Of the forest-interior species, one was found in 15 (50%), four in >2 (≥10%), and seven in ≤2 (<10%) young stands. The number of substrates occupied by different forest-interior species in the young stands were between 1 and 28 (n = 2 843).

### Species composition

**Figure 2:**
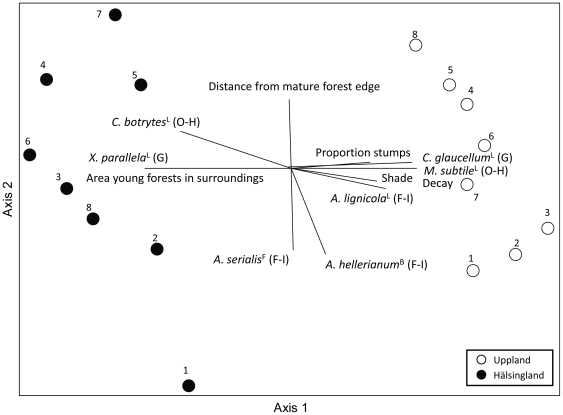
Epixylic cryptogam species composition in young stands along the distance from the mature stand. Non-metric multidimensional scaling (NMS) graph, where circles respresent transects (1–8) in Uppland (open) and Hälsingland (filled). Average values of individual species occupancy (arcsine squareroot transformed) in each transect number and each region were used in the calculations of ordination scores. Lines indicate the direction and strength (line length) of correlations (|r| ≥ 0.60) between ordination scores and explanatory variables (including transformed average occupancy values for individual species). L = lichen, B = bryophyte, F = fungus, O-H = open-habitat, F-I = forest-interior, G = generalist. Axis 1 and 2 explained 82 and 11% of the variance in the data, respectively.

The species composition of epixylic cryptogams in the young stands was affected by distance from the mature forest: transect one (closest to the mature forest) differed from all other transects except number eight, and transect two differed from transect four and six (Table 1). The NMS-ordination revealed that edge distance (r = 0.67) and the occupancy of one open-habitat species (*C. botrytes*, r = 0.49) were positively correlated with NMS-axis two, whereas the occupancies of two forest-interior (*A. hellerianum*, r = −0.74; *A. serialis*, r = −0.73) species were negatively correlated with axis two (Fig. 2). Region also affected cryptogam species composition, as transects from the two study regions were clearly separated along ordination axis one (Fig. 2). The variables with the strongest correlation with axis one were area of young forests in the surroundings (r = 0.97), decay (r = −0.90), and shade (r = −0.74).

**Table 1:** Score statistics from randomized complete blocks PERMANOVA (permutational multivariate analysis of variance) of species composition along the distance from the mature forest.

Source	df	MS	F	*p*	Pairwise comparisons (*p*<0.05) for
					transect number (edge distance)
Stand	21	0.489	5.52	<0.001	
Edge distance	7	0.134	1.51	0.033	1 ≠ 2, 3, 4, 5, 6, 7; 2 ≠ 4*, 6
Residual	147	0.089			
Total	175				

*p = 0.05.

### Effects of distance from the mature forest on species occupancy

**Figure 3:**
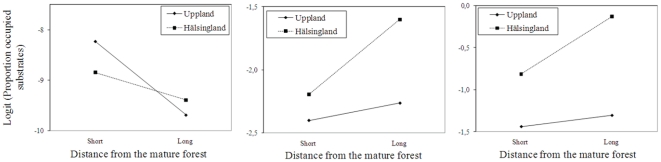
Interactions between explanatory variables. Plots for effects of the interaction between region and distance from the mature forest (“short” = transect 1, and “long” = transect 8) on occupancy of a) forest-interior species, b) open-habitat species, and c) *C. botrytes* (open-habitat lichen). The plots are based on parameter estimates in generalized linear mixed models for the two variables and the intercept of the final model (following http://www.jeremydawson.co.uk/slopes.htm, accessed september 2010).

With increasing distance from the mature forest the occupancy decreased for forest-interior, increased for open-habitat, but was unaffected for generalist cryptogam species (Table 2). In addition, the plots for the interaction between region and distance reveal that with increasing distance the decrease in occupancy for forest-interior species was steeper in Uppland, whereas for open-habitat species the increase in occupancy was steeper in Hälsingland (Fig. 3a, b).

**Table 2:** Parameter estimates (Est.) in generalized linear mixed models for within-transect occupancy of at least one forest-interior, open-habitat or generalist cryptogam species.

Variable	Forest-interior				Open-habitat				Generalist			
	Est.	SE	df	*p*	Est.	SE	df	*p*	Est.	SE	df	*p*
Intercept	−7.60	1.32	28	***	−2.46	0.92	28	0.01	−2.39	0.57	29	***
Region^R^	1.01^U^	0.48	28	0.05	0.01	0.42	28	0.98				
Edge												
distance	−0.32	0.08	206	***	0.03	0.03	203	***				
Proportion												
stumps					4.69	0.86	203	***				
Area^A^	0.90	0.25	206	***					0.26	0.14	209	0.06
Shade	0.95	0.40	206	0.02	−0.43	0.22	203	0.05				
Decay					0.07	0.50	203	0.89				
Decay^2^					0.29	0.08	203	***				
Region×												
edge dist.	0.20	0.11	206	0.07	0.10	0.04	203	0.01				
Prop. stu. x												
decay					−1.77	0.47	203	***				

R Significant estimate followed by H or U denote higher probability of occurrence in Hälsingland or Uppland, respectively.

A Area = log(Average substrate area).

*** = *p*<0.001.

For individual species that were affected by distance from mature forest the qualitative effect was opposite for forest-interior and open-habitat species (Table S3): occupancy decreased for two forest-interior species (*A. lignicola*, and *A. hellerianum*) but increased for one open-habitat species (*C. botrytes*) with increasing distance from the mature forest. The increase in occupancy for *C. botrytes* (open-habitat species) with increasing distance was steeper in Hälsingland than in Uppland (Fig. 3c). No generalist species was affected by distance. The additional analyses of occurrence probability of individual species on individual substrates showed similar results as the transect-level analyses: occurrence probability decreased for the two forest-interior species (*A. lignicola*: estimate = −0.42, *p*<0.001; *A. hellerianum*: estimate = −0.46, *p* = 0.02) but increased for the open-habitat species (*C. botrytes*: estimate = 0.03, *p*<0.001) with increasing distance from the mature forest.

### Effects of local variables and propagule sources on species occupancy

Increasing shade had opposite effects on forest-interior and open-habitat species, with an increase in occupancy for the former and a decrease for the latter (Table 2). Increasing proportion of stumps within transects increased occupancy for open-habitat species but did not affect forest-interior or generalist species. There were some significant interactions in the models. For instance, the occupancy for open-habitat species increased with increasing decay if the proportion of stumps within transects decreased, but decreased with increasing decay if the proportion of stumps increased (Figure S1). In addition, the positive parameter estimate for decay squared, suggests that occupancy for open-habitat species increased curvilinearly when both logs and stumps were considered together. Finally, increasing average substrate area of in the transect increased occupancy for both forest-interior and generalist species.

A larger set of variables explained the occupancy for individual species, but the qualitative effect of variables that explained occurrences of both individual species and the species group to which it belonged was similar (Table S3). Consequently, occupancy for the forest-interior lichen *A*. *lignicola* increased with increasing shade, but decreased for the open-habitat lichen *M*. *subtile*. In addition, occupancy for the two open-habitat lichens *C. botrytes* and *M. subtile* increased with increasing proportion of stumps within transect. However, occupancy for the two forest-interior fungi *A*. *serialis* and *T*. *abietinum* also increased with increasing proportion of stumps. The significant interaction between proportion stumps within transects and decay for the open-habitat lichen *M*. *subtile* suggests further that occupancy for this species increased with increasing decay if the proportion of stumps within transects decreased, but decreased with increasing decay if the proportion of stumps increased (Figure S1).

The parameter estimates for decay and decay squared indicate that occupancy for the open-habitat lichen *C*. *botrytes* decreased non-linearly with increasing decay and that two forest-interior species (*A*. *lignicola* and *A*. *hellerianum*) showed a maximum at intermediate decay (Table S3). Increasing average substrate area of in the transect increased occupancy for three forest-interior (*A*. *hellerianum*, *A*. *serialis*, and *T*. *abietinum*), one open-habitat (*M. subtile*), and two generalist species (*C. glaucellum* and *X. parallela*). For the two forest-interior fungi *A*. *serialis*, and *T*. *abietinum*, there was, however, a positive effect of increasing substrate area of only with decreasing proportion of stumps within transects (Figure S1). In contrast, the occupancy for the generalist lichen *X. paralella* increased with increasing area only with increasing proportion of stumps within transects. The occupancy for the generalist lichens *C*. *trabinellum* and *X*. *vitiligo* was not related to any of the variables tested. Finally, the predictors for local or landscape level propagule availability did not contribute to any model.

## Discussion

We show that at least some forest-interior epixylic lichens, bryophytes, and fungi are indeed able to both colonize and survive in matrix habitats such as young clear-cut boreal forests. As hypothesized, the occupancy of forest-interior species was higher near the edge to the mature forest (positive edge effect in the matrix), whereas open-habitat species (where the conclusions, strictly speaking, should be restricted to lichens) were less frequently encountered in the vicinity of the mature forest (negative edge effect). Substrate area was the only significant predictor for the occupancy of generalist species. This may be a direct area effect, reducing local extinctions [42], but could also reflect the larger range of microhabitat conditions on larger substrates.

Our results contrast with the findings of Hylander [43], and Rudolphi and Gustafsson [12], who did not find any edge effects on the re-colonization patterns of ground-living forest-interior bryophytes, or epiphytic and epixylic forest-interior lichens and bryophytes in young forest stands (matrix habitats) bordering mature focal habitats. The matrix habitats in their studies were, however, older than ours (mean age>40 years, our mean age = 11 years), and biological edge effects are likely to diminish as the young stands mature and the structural differences between stand types decrease [2], [30]. In addition, edge effects due to dispersal limitations between stands will probably also decrease with increasing time that a substrate has been available for colonization. In accordance with the studies mentioned above, though, we did not find an effect of local propagule source.

### Effects of distance from the mature forest – potential mechanisms

We envision a number of possible mechanisms behind the observed positive and negative edge effects, given the spatio-temporal scale of the study, and the following discussion could be viewed as a basis for hypotheses that could be tested by e.g. fine-scale population monitoring or experiments.

The gradual increase in exposure to sun and wind in young stands with increasing distance from mature forest edges [20] is likely to explain the effects of edge distance on forest-interior and open-habitat species. Cryptogams lack the ability to regulate water uptake and loss, and are known to respond to microclimate [25]–[28], [44]. The drier conditions towards the core of young stands may, thus, decrease the probability of survival and colonization of forest-interior cryptogams after clear-cut disturbance, whereas the more moist microclimate and higher water content of the substrates in the less exposed young forest edge, may be less suitable for open-habitat species. In addition, the quantitative differences in the observed edge effects between regions may be due to differences in the species pool (as revealed by the multivariate analyses), climate and forest history.

Local environmental variables (decay, shade, type, and substrate area) also affected the occurrence of the epixylic cryptogam species in our study, but they did not correlate with distance to the mature forest. Hence, the spatial distribution of substrates of different qualities cannot have caused the observed edge effects on forest-interior and open-habitat species. Even though our measure of shade is rather crude and assesses the conditions at the time of sampling it was the single most important local variable explaining differences in occupancy between forest-interior and open-habitat species. As expected, there was a positive effect on the former and a negative effect on the latter. Sheltered, small-scale refugia have been shown to increase the survival of ground-living forest-interior bryophytes following clear-cutting [11]. In addition, forest-interior fungi may respond with a time lag to disturbance and survive under unfavourable microclimatic conditions [28]. By judging log age and population size and growth rate, we suggest that also one epixylic forest-interior bryophyte (A. hellerianum) can survive the clear-cutting disturbance: highly decayed logs (decay classes 4 and 5, representing 5% of all logs) most likely originated from the forest before logging, and two occurrences of A. hellerianum were recorded on such logs. Substrates that are created and colonized before clear-cutting could, hence, be a potential internal propagule source for the recovery of epixylic forest-interior species in young stands, especially if they are protected against desiccation by the shade of surrounding trees and bushes.

That forest-interior fungi can survive under unfavourable microclimatic conditions [28] could explain why two forest-interior fungi (A. serialis and T. abietinum) were not affected by distance to the mature forest, even if the mature forest is their ‘preferred’ habitat (judging from the higher occurrences). It could be that these two species are more of substrate specialists than habitat specialists. We therefore made a simple test of the sensitivity of our results to their classification: treating them as generalists did not change the effect of distance on the forest-interior species as a group, nor did it change the results for the generalist species group.

The occurrence patterns of the forest-interior cryptogam species in our study was not affected by local propagule sources (i.e. from the bordering focal habitat). This does not necessarily mean that the studied species do not have restricted dispersal, which has been shown on local scales for lichens [31], bryophytes [32], and fungi [33]. Possible dispersal limitations between stand types could simply be masked by the importance of internal propagule sources, and the observed positive edge effects on forest-interior species could reflect a pattern of survival rather than of colonization, or a combination of both. Sufficient regional propagule rain (i.e. on a larger spatial scale than what is accounted for in our study) for forest-interior cryptogams may also decrease the relative importance of local propagule sources, as has been suggested for the recovery of ground-living bryophytes in young stands [43]. Both internal and external (local as well as regional) propagule sources are, thus, likely to affect colonization by forest-interior species after clear-cut disturbance, and restricted dispersal between stands does, therefore, not seem to explain the observed positive edge effects. Neither is it likely that the negative edge effects on open-habitat species are due to dispersal limitations within stands since their propagule sources should be open habitats in the surroundings. These species were, however, not affected by amount of young forests within a radius of 500 m, suggesting that there are sufficient propagule sources of open-habitat cryptogams on a larger spatial scale than 500 m in this managed forest landscape. We conclude that the variation in the studied species assemblage along with distance from the mature forest, is better explained by microclimatic gradients (acting directly or by altering competitive abilities) than by dispersal limitations from local propagule sources.

### Implications for conservation of forest-interior cryptogams

In addition to previous recommendations that stressed the need for greater conservation efforts only at the south side of retained boreal forest stands [27], we suggest that retention and creation of dead wood in managed clear-cut forests at the north side of retained mature forests is a feasible way to enhance re-establishment of forest-interior epixylics (including rare and red-listed species) in regenerating young boreal forest stands. These efforts should have a greater positive effect on epixylic cryptogams if dead wood is supplied not only once, but throughout the maturation of the regenerating forest, as this may enhance resilience on the landscape level. In addition, since edge effects probably will diminish as the matrix habitat matures and the structural differences between the stand types decrease [2], [30], we propose that creation and retention of dead wood should be performed in the whole stand as it matures. Efficient strategies for preserving epixylic forest-interior species in managed forest landscapes in the northern hemisphere can be to avoid to harvest logging residues (branches, tops, and stumps) for biofuel in clear-cuts along the southern edges to mature forests, and to leave dead wood created at clearing and thinning operations in stands located to the north of old-growth stands. Since microclimate was of greater importance than dispersal limitation for the occurrence of epixylic forest-interior cryptogams in the young stands, it still remains to be tested whether these species may occur in young managed boreal stands located south of old-growth forests, i.e. where there is a sharp contrast in microclimate between stand types.

### Conclusions

Many conservation issues have been described in terms of focal habitats in a matrix, and most management suggestions have focused on how to decrease the negative impacts of edge effects from matrix habitats on focal habitats. We argue that management with the aim to increase habitat quality in matrix habitats bordering focal habitats should increase the probability of long-term persistence of habitat specialists. Thus, in addition to the protection and management of the few old-growth forests in fragmented forest landscapes, management actions should also be directed to matrix habitats such as young forests.

## Supporting Information

Table S1 Species classification and occurences of study species at the stand and substrate level.
(DOC)

Table S2 Mean and range of explanatory variables used in the generalized linear mixed models.
(DOC)

Table S3 Parameter estimates in generalized linear mixed models for within-transect occupancy of individual lichen, bryophyte and fungal species.
(DOC)

Figure S1 Plots for effects of interactions between variables in generalized linear models. Interaction between decay and proportion of stumps within transects on occupancy of a) open-habitat species, and b) *M. subtile* (open-habitat lichen), and for effects of the interaction between substrate area and proportion of stumps within transects on occupancy of c) *A. serialis* (forest-interior fungi), d) *T. abietinum* (forest-interior fungi), and e) *X. parallela* (generalist lichen). Plots are based on logistic regressions with only the x- and y-axis variables, and their interaction.
(TIF)
