# Supplementary material for: Positive Edge Effects on Forest-Interior Cryptogams in Clear-Cuts
Source: PLoS One. 2011 Nov 17;6(11):e27936. doi: 10.1371/journal.pone.0027936 (PMC3219701; doi:10.1371/journal.pone.0027936)
Supplement: Table S1 — Species classification and occurences of study species at the stand and substrate level. (DOC) [file pone.0027936.s001.doc]

Table S1. Occurences of study species at the stand and substrate level. Species classification according to expert evaluations (A Dahlberg - fungi, T Hallingbäck - bryophytes, and G Thor – lichens) and Hallingbäck [1], Hallingbäck [2], and Hallingbäck and Aronsson [3]. Red list category according to the Red List of the Swedish Species [4]: NT = near threatened, VU = vulnerable, and EN = endangered. The nomenclature follows Santesson et al. [5] for lichens (except that *Lecidea gibberosa* coll. also include *L*. *symmictella*), Hallingbäck et al. [6] for bryophytes, and Ryman and Holmåsen [7] for fungi.

| Cryptogam species | No. mature stands  (n = 30) | No. young stands  (n = 30) | No. occupied objects  (n = 2 843) | Red list category |
| --- | --- | --- | --- | --- |
| Forest-interior |  |  |  |  |
| *Absconditella lignicola*^L^ | 4 | 7 | 28 | - |
| *Chaenotheca gracillima*^L^ | 6 | 0 | 0 | - |
| *Chaenotheca laevigata*^L^ | 2 | 0 | 0 | - |
| *Anastrophyllum hellerianum*^B^ | 25 | 8 | 11 | NT |
| *Anastrophyllum michauxii*^B^ | 0 | 0 | 0 | NT |
| *Buxbaumia viridis*^B^ | 4 | 0 | 0 | - |
| *Calypogeia suecica*^B^ | 11 | 1 | 1 | VU |
| *Cephalozia catenulata*^B^ | 3 | 0 | 0 | - |
| *Cephalozia lunulifolia*^B^ | 6 | 0 | 0 | - |
| *Hypnum pallescens*^B^ | 0 | 0 | 0 | - |
| *Lophozia ascendens*^B^ | 0 | 0 | 0 | VU |
| *Lophozia longiflora*^B^ | 1 | 1 | 1 | NT |
| *Nowellia curvifolia*^B^ | 2 | 1 | 1 | - |
| *Riccardia latifrons*^B^ | 4 | 2 | 2 | - |
| *Riccardia palmata*^B^ | 2 | 2 | 2 | - |
| *Scapania umbrosa*^B^ | 0 | 0 | 0 | - |
| *Tritomaria exsecta*^B^ | 0 | 0 | 0 | EN |
| *Amylocystis lapponica*^F^ | 0 | 0 | 0 | VU |
| *Antrodia serialis*^F^ | 21 | 15 | 23 | - |
| *Asterodon ferruginosus*^F^ | 4 | 1 | 1 | NT |
| *Climacocystis borealis*^F^ | 1 | 0 | 0 | - |
| *Columnocystis abietina*^F^ | 0 | 0 | 0 | - |
| *Fomitopsis rosea*^F^ | 5 | 0 | 0 | - |
| *Inonotus leporinus*^F^ | 0 | 0 | 0 | NT |
| *Junghuhnia collabens*^F^ | 0 | 0 | 0 | VU |
| *Phellinus chrysoloma*^F^ | 3 | 2 | 2 | - |
| *Phellinus ferrugineofuscus*^F^ | 10 | 0 | 0 | - |
| *Phellinus nigrolimitatus*^F^ | 0 | 0 | 0 | NT |
| *Phellinus viticola*^F^ | 12 | 5 | 5 | - |
| *Phlebia centrifuga*^F^ | 2 | 0 | 0 | - |
| *Stereum sanguinolentum*^F^ | 0 | 0 | 0 | - |
| *Trichaptum abietinum*^F^ | 24 | 9 | 16 | - |

^L^ Lichen, ^B^ Bryophyte, ^F^ Fungus

Appendix A. Continued.

|  | No. mature stands  (n = 30) | No. young stands  (n = 30) | No. occupied objects  (n = 2 859) | Red list category |
| --- | --- | --- | --- | --- |
| Open-habitat |  |  |  |  |
| *Calicium abietinum*^L^ | 0 | 0 | 0 | VU |
| *Calicium denigratum*^L^ | 2 | 0 | 0 | - |
| *Cladonia botrytes*^L^ | 12 | 30 | 1034 | - |
| *Cladonia parasitica*^L^ | 1 | 1 | 1 | NT |
| *Cyphelium inquinans*^L^ | 0 | 0 | 0 | - |
| *Cyphelium tigillare*^L^ | 0 | 0 | 0 | NT |
| *Lecidea gibberosa* coll*.* ^L^ | 5 | 18 | 62 | - |
| *Mycocalicium subtile*^L^ | 26 | 25 | 283 | - |
| *Pyrrhospora elabens*^L^ | 0 | 1 | 1 | - |
| Generalists |  |  |  |  |
| *Calicium glaucellum*^L^ | 26 | 15 | 54 | - |
| *Calicium trabinellum*^L^ | 5 | 5 | 10 | - |
| *Chaenotheca chlorella*^L^ | 0 | 1 | 1 | - |
| *Hypocenomyce antracophila*^L^ | 5 | 2 | 2 | - |
| *Hypocenomyce castaneocinerea*^L^ | 4 | 2 | 3 | NT |
| *Lecidea botryosa*^L^ | 3 | 5 | 7 | - |
| *Xylographa parallela*^L^ | 13 | 29 | 551 | - |
| *Xylographa vitiligo*^L^ | 15 | 17 | 49 | - |

^L^ Lichen, ^B^ Bryophyte, ^F^ Fungus

**References**

1. Hallingbäck T (1995) Ekologisk katalog över lavar. [The lichens of Sweden and their ecology] ArtDatabanken, SLU. Uppsala.
2. Hallingbäck T (1996) Ekologisk katalog över mossor. [The bryophytes of Sweden and their ecology.] ArtDatabanken, SLU. Uppsala.
3. Hallingbäck T, Aronsson G (1998) Ekologisk katalog över storsvampar och myxomyceter. [Macrofungi and myxomycetes of Sweden and their ecology] ArtDatabanken, SLU. Uppsala.
4. Gärdenfors U (2010) Rödlistade arter i Sverige 2010 - The 2010 Red List of Swedish Species. Swedish Species Information Centre, SLU, Uppsala, Sweden.
5. Santesson R, Moberg R, Nordin A, Tönsberg T, Vitikainen O (2004) Lichen-forming and lichenicolous fungi of Fennoscandia. Museum of Evolution, Uppsala University, Uppsala.
6. Hallingbäck T, Hedenäs L, Weibull H (2006) Ny checklista för Sveriges mossor. Svensk botanisk tidskrift 100: 96-148.
7. Ryman S, Holmåsen I (1992) Svampar - En fälthandbok. Interpublishing AB, Stockholm.
