# Supplementary material for: Positive Edge Effects on Forest-Interior Cryptogams in Clear-Cuts
Source: PLoS One. 2011 Nov 17;6(11):e27936. doi: 10.1371/journal.pone.0027936 (PMC3219701; doi:10.1371/journal.pone.0027936)
Supplement: Table S2 — Mean and range of explanatory variables used in the generalized linear mixed models. (DOC) [file pone.0027936.s002.doc]

Table s2. Mean and range of explanatory variables used in the generalized linear mixed models for within-transect occupancy of species groups and individual cryptogam species.

| Explanatory variable | Mean | Min | Max |
| --- | --- | --- | --- |
| Stand variables |  |  |  |
| Stand age | 11 | 6 | 21 |
| Number of forest-interior species in mature stand | 4.9 | 0 | 9 |
| Area young stands within 500 m (ha) | 7.6 | 0 | 29 |
| Local variables |  |  |  |
| Substrate area (dm^2^) | 55 | 19 | 247 |
| Proportion stumps within transects | 0.8 | 0.2 | 1 |
| Decay | 1.9 | 1 | 5 |
| Shade | 1.8 | 1 | 3 |
