# Supplementary material for: Positive Edge Effects on Forest-Interior Cryptogams in Clear-Cuts
Source: PLoS One. 2011 Nov 17;6(11):e27936. doi: 10.1371/journal.pone.0027936 (PMC3219701; doi:10.1371/journal.pone.0027936)
Supplement: Table S3 — Parameter estimates in generalized linear mixed models for within-transect occupancy of individual lichen, bryophyte and fungal species. (DOC) [file pone.0027936.s003.doc]

Table S3. Parameter estimates (Est.) in generalized linear mixed models for within-transect occupancy of individual lichen (L), bryophyte (B) and fungal (F) species.

|  |  | **Intercept** | **Region^R^** | **Edge**  **distance** | **Proportion**  **stumps** | **Area^A^** | **Shade** | **Decay** | **Decay^2^** | **Region x Edge**  **distance** | **Prop. stumps x Decay** | **Prop. stumps x Area^A^** | |
| --- | --- | --- | --- | --- | --- | --- | --- | --- | --- | --- | --- | --- | --- |
| *Absconditella* | Est. | -11.97 | 2.34^U^ | -0.35 |  |  | 1.57 | 4.02 | -0.66 |  |  |  | |
| *linicola*^L^ | SE | 3.28 | 1.27 | 0.12 |  |  | 0.92 | 2.22 | 0.39 |  |  |  | |
| (28*) | df | 28 | 28 | 206 |  |  | 206 | 206 | 206 |  |  |  | |
| For.-interior | *p* | 0.001 | 0.076 | 0.004 |  |  | 0.089 | 0.071 | 0.087 |  |  |  | |
| *Anastrophyllum* | Est. | -23.70 |  | -0.49 |  | 2.34 |  | 8.36 | -1.57 |  |  |  | |
| *hellerianum*^B^ | SE | 5.29 |  | 0.21 |  | 0.61 |  | 3.34 | 0.69 |  |  |  | |
| (11*) | df | 29 |  | 206 |  | 206 |  | 206 | 206 |  |  |  | |
| For.-interior | *p* | *** |  | 0.019 |  | *** |  | 0.013 | 0.024 |  |  |  | |
| *Antrodia* | Est. | -18.93 |  |  | 13.05 | 3.02 |  |  |  |  |  | -2.66 | |
| *serialis*^F^ | SE | 6.78 |  |  | 7.88 | 1.45 |  |  |  |  |  | 1.79 | |
| (23*) | df | 29 |  |  | 207 | 207 |  |  |  |  |  | 207 | |
| For.-interior | *p* | 0.009 |  |  | 0.099 | 0.038 |  |  |  |  |  | 0.138 | |
| *Trichaptum* | Est. | -18.06 |  |  | 18.40 | 3.78 |  |  |  |  |  | -5.62 | |
| *abietinum*^F^ | SE | 6.20 |  |  | 8.77 | 1.31 |  |  |  |  |  | 2.08 | |
| (16*) | df | 29 |  |  | 207 | 207 |  |  |  |  |  | 207 | |
| For.-interior | *p* | 0.007 |  |  | 0.037 | 0.004 |  |  |  |  |  | 0.007 | |
| *Cladonia* | Est. | -1.50 | 0.39 | 0.03 | 1.87 |  |  | -1.31 | 0.23 | 0.12 |  |  | |
| *botrytes*^L^ | SE | 0.64 | 0.49 | 0.03 | 0.34 |  |  | 0.38 | 0.08 | 0.04 |  |  | |
| (1034*) | df | 28 | 28 | 205 | 205 |  |  | 205 | 205 | 205 |  |  | |
| Open-habitat | *p* | 0.026 | 0.438 | *** | *** |  |  | *** | 0.003 | 0.003 |  |  | |
| *Mycocalicium* | Est. | -6.98 | 1.77^U^ |  | 4.96 | 0.59 | -0.82 | 1.70 |  |  | 1.90 | |  |
| *subtile*^L^ | SE | 1.87 | 0.40 |  | 1.47 | 0.24 | 0.31 | 0.60 |  |  | 0.65 | |  |
| (283*) | df | 28 | 28 |  | 205 | 205 | 205 | 205 |  |  | 205 | |  |
| Open-habitat | *p* | *** | *** |  | *** | 0.017 | 0.009 | 0.005 |  |  | 0.004 | |  |

^*^ Number of occurrences observed, n = 2 843.

^R^ Significant estimate followed by H or U denote higher probability of occurrence in Hälsingland or Uppland, respectively.

^A^ Area = log(Average substrate area).

*** = *p*<0.001

Table 3. Continued.

|  |  | **Intercept** | **Region^R^** | **Edge**  **distance** | **Proportion**  **stumps** | **Area^A^** | **Shade** | **Decay** | **Decay^2^** | **Region x Edge**  **distance** | **Prop. stumps x Decay** | **Prop. stumps x Area^A^** |
| --- | --- | --- | --- | --- | --- | --- | --- | --- | --- | --- | --- | --- |
| *Calicium* | Est. | -8.00 | 3.09^U^ |  |  | 1.13 |  |  |  |  |  |  |
| *glaucellum*^L^ | SE | 1.50 | 0.76 |  |  | 0.35 |  |  |  |  |  |  |
| (54*) | df | 28 | 28 |  |  | 209 |  |  |  |  |  |  |
| Generalist | *p* | *** | *** |  |  | 0.002 |  |  |  |  |  |  |
| *Xylographa* | Est. | 0.05 | 1.01^H^ |  | -4.56 | -0.62 |  |  |  |  |  | 1.26 |
| *parallela*^L^ | SE | 2.44 | 0.36 |  | 2.89 | 0.57 |  |  |  |  |  | 0.71 |
| (551*) | df | 28 | 28 |  | 207 | 207 |  |  |  |  |  | 207 |
| Generalist | *p* | 0.983 | 0.009 |  | 0.116 | 0.279 |  |  |  |  |  | 0.080 |

^*^ Number of occurrences observed, n = 2 843.

^R^ Significant estimate followed by H or U denote higher probability of occurrence in Hälsingland or Uppland, respectively.

^A^ Area = log(Average substrate area).

*** = *p*<0.001
