# Supplementary figures and images for: Positive Edge Effects on Forest-Interior Cryptogams in Clear-Cuts
Source: PLoS One. 2011 Nov 17;6(11):e27936. doi: 10.1371/journal.pone.0027936 (PMC3219701; doi:10.1371/journal.pone.0027936)

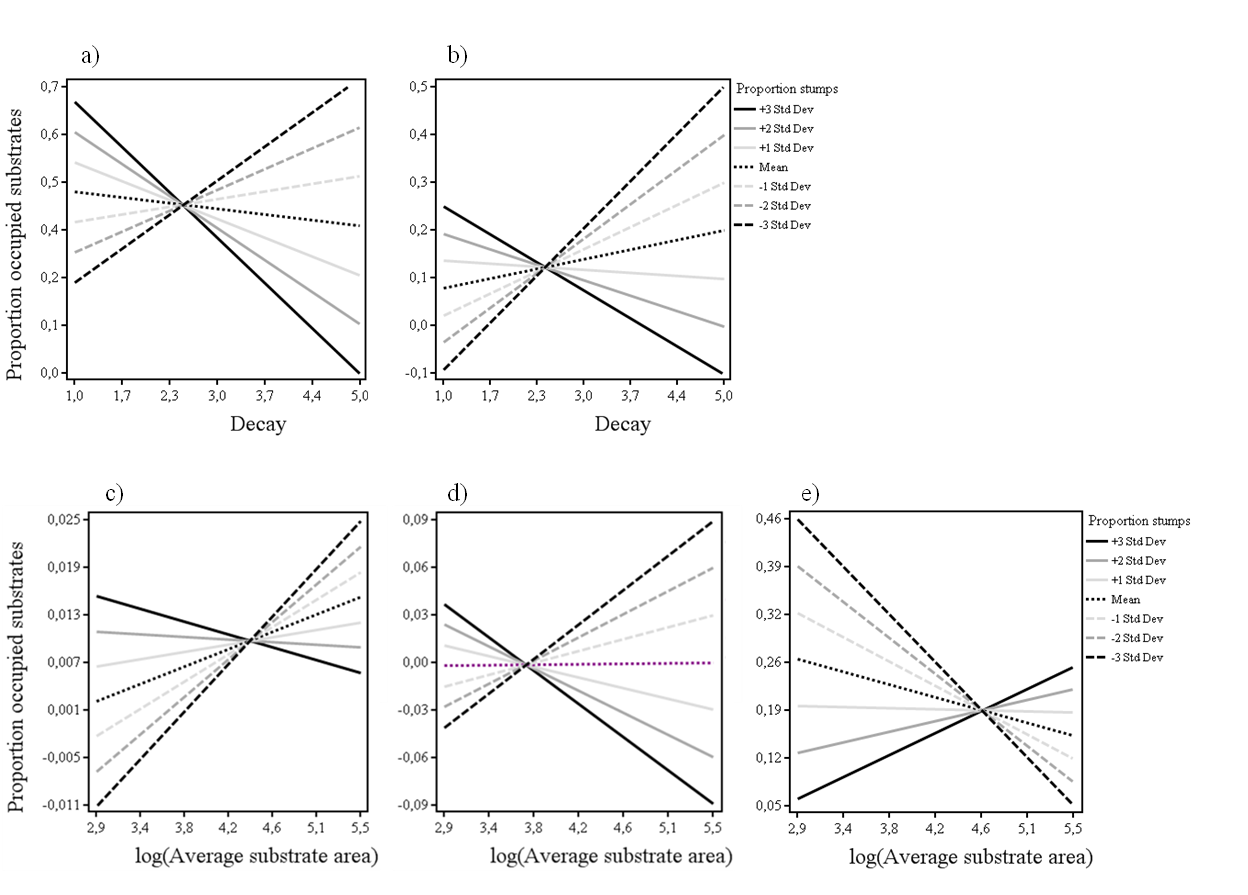

Supplement: Figure S1 — Plots for effects of interactions between variables in generalized linear models. Interaction between decay and proportion of stumps within transects on occupancy of a) open-habitat species, and b) M. subtile (open-habitat lichen), and for effects of the interaction between substrate area and proportion of stumps within transects on occupancy of c) A. serialis (forest-interior fungi), d) T. abietinum (forest-interior fungi), and e) X. parallela (generalist lichen). Plots are based on logistic regressions with only the x- and y-axis variables, and their interaction. (TIF) [file pone.0027936.s004.tif]
